# Biomimetic Mineralization Promotes Viability and Differentiation of Human Mesenchymal Stem Cells in a Perfusion Bioreactor

**DOI:** 10.3390/ijms22031447

**Published:** 2021-02-01

**Authors:** Gloria Belén Ramírez-Rodríguez, Ana Rita Pereira, Marietta Herrmann, Jan Hansmann, José Manuel Delgado-López, Simone Sprio, Anna Tampieri, Monica Sandri

**Affiliations:** 1 BioNanoMet Group, Department of Inorganic Chemistry, University of Granada, 18071 Granada, Spain; jmdl@ugr.es; 2 IZKF Group Tissue Regeneration in Musculoskeletal Diseases, University Hospital Wuerzburg, 97070 Wuerzburg, Germany; r-pereira.klh@uni-wuerzburg.de (A.R.P.); m-herrmann.klh@uni-wuerzburg.de (M.H.); jan.hansmann@uni-wuerzburg.de (J.H.); 3 Bernhard-Heine-Centrum for Locomotion Research, University of Wuerzburg, 97070 Wuerzburg, Germany; 4 Institute of Science and Technology for Ceramics (ISTEC-CNR), 48018 Faenza, Italy; simone.sprio@istec.cnr.it (S.S.); anna.tampieri@istec.cnr.it (A.T.); monica.sandri@istec.cnr.it (M.S.)

**Keywords:** scaffold, perfusion bioreactor, collagen, apatite nanoparticles, magnesium, human mesenchymal stem cell, osteogenesis

## Abstract

In bone tissue engineering, the design of 3D systems capable of recreating composition, architecture and micromechanical environment of the native extracellular matrix (ECM) is still a challenge. While perfusion bioreactors have been proposed as potential tool to apply biomechanical stimuli, its use has been limited to a low number of biomaterials. In this work, we propose the culture of human mesenchymal stem cells (hMSC) in biomimetic mineralized recombinant collagen scaffolds with a perfusion bioreactor to simultaneously provide biochemical and biophysical cues guiding stem cell fate. The scaffolds were fabricated by mineralization of recombinant collagen in the presence of magnesium (RCP.MgAp). The organic matrix was homogeneously mineralized with apatite nanocrystals, similar in composition to those found in bone. X-Ray microtomography images revealed isotropic porous structure with optimum porosity for cell ingrowth. In fact, an optimal cell repopulation through the entire scaffolds was obtained after 1 day of dynamic seeding in the bioreactor. Remarkably, RCP.MgAp scaffolds exhibited higher cell viability and a clear trend of up-regulation of osteogenic genes than control (non-mineralized) scaffolds. Results demonstrate the potential of the combination of biomimetic mineralization of recombinant collagen in presence of magnesium and dynamic culture of hMSC as a promising strategy to closely mimic bone ECM.

## 1. Introduction

Severe trauma, congenital malformations or disease can compromise the integrity and functionality of the skeletal system to the extent of requiring bone implants [1]. Over 2 million bone surgeries are conducted each year in the Unites States and around 10 million of Chinese patients suffered from limited limb functions [2]. Bone is the second most transplanted tissue after blood and an ever-increasing worldwide demand of bone graft substitutes can be expected in the coming years [1]. The forefront of tissue engineering involves the isolation of stem cells from bone marrow or adipose tissue aspirated from the patient, their expansion and differentiation on synthetic porous scaffolds and the further implantation of the composite with the aim of fostering osteoinduction and osteoconduction for rapid repair of the defect site [3,4]. The design of ideal bone biomaterial plays a vital role in bone repair. Biomaterial design aims to recapitulate the organization and composition of natural bone extracellular matrix (ECM) where stem cells grow in vivo. To guide stem cell interaction and fate, multiple stimulus including physical (porosity, surface nanotopography, stiffness, shear stress and electrical forces) and biochemical (surface chemistry, growth factors o proteins) have been proposed [2,5,6,7].

In vivo bone mineralization has inspired the design of advanced biomaterials, so-called biomimetic, mimicking the hierarchical organization and composition of native tissue at nanoscale level [8,9,10,11]. Bone is a nanocomposite made up of self-assembled collagen fibers (accounting for 35 wt.%) strengthened by inter and intrafibrillar mineralization of apatite (Ap) nanocrystals (65 wt.%) [12]. Ap nanoparticles consist on non-stoichiometric hydroxyapatite [HA, Ca_10_(PO_4_)_6_(OH)_2_] containing foreign ions in its structure, e.g., CO_3_^2−^, Mg^2+^, Sr^2+^ [13]. Bone grafts have been designed through a biologically inspired mineralization protocol by which magnesium-substituted hydroxyapatite nanoparticles crystallized into naturally derived type I collagen fibers mimicking the process occurring during biological neo-ossification [9,14,15,16,17]. In vitro studies demonstrated the potential of this hybrid scaffolds to induce, orchestrate, and harmonize the osteogenic differentiation of human bone marrow stem cells (hMSCs) and in vivo studies revealed its osteoinductive nature [17]. However, the difficulties in processing and purification with batch-to-batch variability and the risk of disease transmission of natural collagen have stimulated keen interest in devising biomaterial based on recombinant collagen [18,19]. Recombinant collagen is engineered with precise chemical composition and molecular weight, enabling the introduction of specific amino acid sequences and the large-scale production for industrial exploitation [18]. Recently, we have investigated the biomimetic mineralization of recombinant collagen peptide (RCP) based on human collagen type I enriched in arginine-glycine-aspartic acid sequences, which are specific sites favoring the attachment of a large number of adhesive ECM, blood and cell surface proteins [20]. We carried out RCP mineralization in the presence of magnesium to closer mimic bone Ap composition (0.5–1%) [21] and we observed that Mg stabilized the precursor amorphous calcium phosphate and inhibited apatite crystal growth along *c*-axis, resulting in more isometric Ap nanocrystals than mineralization protocol without magnesium [20]. Atomic force microscopy observations revealed that mineralization in the presence of Mg provided homogeneous mineral distribution in the organic matrix and surface roughness similar to that found in bone [20]. Further in vitro studies demonstrated that RCP mineralization in the presence of magnesium potentially promoted cell migration through the inner areas of the scaffold and higher gene and protein expressions of osteogenic markers comparing with the results of mouse mesenchymal stem cells grown on mineralized scaffolds without magnesium [22]. In spite of these good results, even after 14 days of cell culture, few cells arrived to the bottom inner part of the scaffolds [22]. This is likely due to the fact that in vitro static cell culture on tridimensional scaffolds presents scarce mass transport prompting to limited cell colonization through few hundred microns and apoptosis or necrosis of cells at the central core of the scaffold [23].

Over the last decade, bioreactor systems have been engineered to improve the transport of gases and nutrients and enhance cell distribution, overcoming the main limitations of static cell culture [7,23,24,25]. Perfusion bioreactors also generate dynamic biophysical stimuli such as shear stress and mechanical loading [7,23,24,25]. Cells in bone tissues (i.e., osteoclast, osteoblast, osteocytes and MSCs) are mechanosensitive and respond to biophysical factors in the environment [23]. It has been widely demonstrated that dynamic cell culture in different type of scaffolds such as ceramic scaffolds, metallic scaffolds, decellularized bone matrix from animal sources, synthetic polymer scaffolds, e.g., poly (l-latic acid), or hybrid scaffolds made of HA and chitosan, enhanced cell proliferation and osteogenic differentiation [26,27]. Several in vivo studies also demonstrated that MSC preconditioning in bioreactor prior to implantation enhanced in vivo results as quantity and quality of new bone [23,28,29]. A more recent work demonstrated that bioreactor-based preconditioning augments the bone-forming potential of human bone marrow aspirates [30].

Despite the numerous works on dynamic culture of MSC in perfusion bioreactor, there is still a lack of simulation of the real environment due to the low biomimicry of used scaffold. In this work, we propose the combination of perfusion bioreactor and recombinant collagen scaffolds mineralized in the presence of magnesium (RCP.MgAp) to simultaneously provide biochemical and biophysical cues and closely simulate the growth environment of stem cells in vivo. The structure and chemical composition of biomimetic scaffold (RCP.MgAp) was deeply characterized. Then, hMSC seeding on the resulting 3D biomaterial was assessed under static and dynamic conditions in a perfusion bioreactor. We also evaluated the osteogenic potential of biomimetic mineralized recombinant collagen scaffolds versus control recombinant collagen scaffolds by gene expression.

## 2. Results

### 2.1. Scaffold Characterization

**Figure 1:**
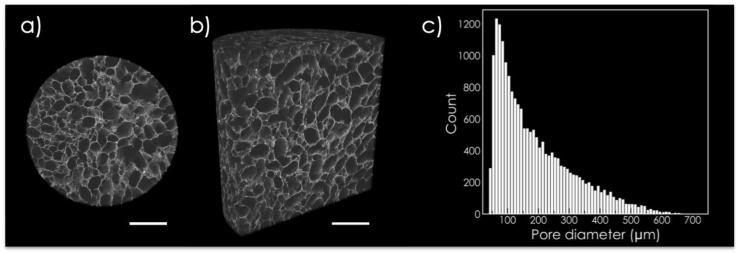
Structural characterization of recombinant collagen in the presence of magnesium (RCP.MgAp) scaffold (diameter = 4 mm; height = 4 mm) by micro-computed tomography. (**a**) Transversal and (**b**) longitudinal section of the scaffold. Scale bar=1 mm. (**c**) Graph bar displays pore size distribution estimated with ImageJ software.

The microstructure of RCP.MgAp scaffold was evaluated via micro-computed tomography (μCT). Tridimensional porous scaffolds with interconnected and randomly distributed pores (i.e., isotropic structure) were obtained via freeze-drying of mineralized solutions (Figure 1). Analysis of surface pore size of 200 μCT scans revealed a wide pore size distribution ranging from 50 to 600 μm and giving rise to 90 ± 0.4% of porosity. Non-mineralized recombinant collagen scaffolds used as a control on in vitro assay showed similar pore size distribution (Figure S1) and porosity (88 ± 0.6%).

**Figure 2:**
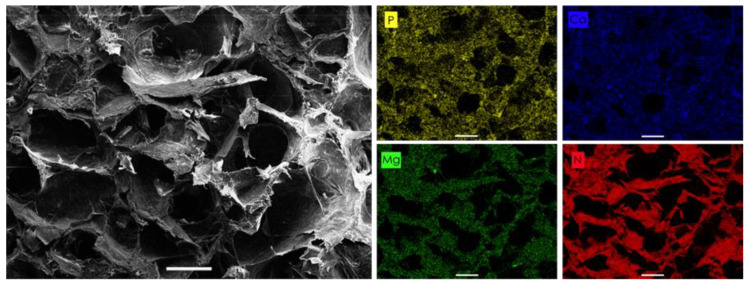
Energy dispersive X-ray spectroscopy (EDS) elemental maps of an area of RCP.MgAp scaffold showing the presence of P (yellow), Ca (blue) and Mg (green) of the mineral phase closely related to the N signal of the collagen (red). SEM image of the scaffold on the left displays the area of the mapping. Scale bar = 100 μm.

**Figure 3:**
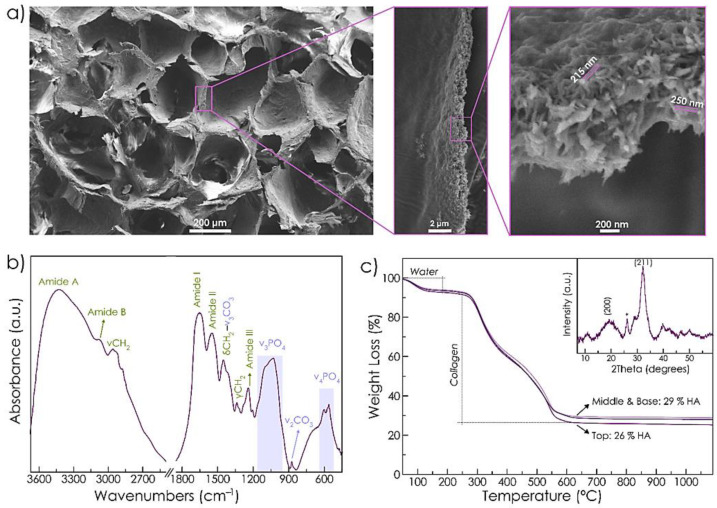
(**a**) SEM images of cross-section of RCP.MgAp scaffold. High magnification image of the wall of the pores shows the incorporation of Ap into the organic matrix. (**b**) Fourier-transform infrared spectroscopy (FTIR) spectrum of RCP.MgAp scaffold. (**c**) TGA curve of the top, middle and base of the scaffold. Inset displays X-ray diffraction (XRD) pattern of the sample exhibiting characteristic reflections of hydroxyapatite (HA, ASTM card file No 09-432).

Scanning electron microscope (SEM) image of RCP.MgAp scaffold shows pores diameter between 100–300 μm (Figure 2). The energy dispersive X-ray spectroscopy (EDS) mapping of the cross-section (Figure 2) reveals a uniform distribution of calcium, phosphorus and magnesium belonging to the mineral phase along with the nitrogen of the organic matrix. From these results we can infer that a homogeneous hybrid material was obtained through the biomimetic mineralization of RCP. In fact, the Ap nanoparticles are completely embedded in the organic matrix as indicated by the SEM images at high magnification of the wall of the pore (Figure 3a). The nanoparticles have a length of approximately 150–250 nm and a width of around 30–50 nm (Figure 3a), as previously observed by transmission electron images of MgAp nanoparticles synthesized through the same biomimetic mineralization approach [20].

The chemical composition and structure of RCP.MgAp scaffolds were also analyzed by Fourier-transform infrared spectroscopy (FTIR), thermogravimetric analysis (TGA) and X-ray diffraction (XRD). FTIR spectrum (Figure 3b) shows the amide bands of recombinant collagen at 1245 cm^−1^ (amide III), 1540 cm^−1^ (amide II), 1650 cm^−1^ (amide I), 3074 cm^−1^ (amide B) and 3421 cm^−1^ (amide A) [31,32]. The wagging (γ), bending (δ) and asymmetrical stretching (ν) modes of CH_2_ of collagen are also visible [31]. It also displays characteristic bands of Ap: the triply degenerated asymmetric stretching mode of phosphate group (ν_3_PO_4_) at 1032 cm^−1^ with shoulders at 1046 cm^−1^ and 1087 cm^−1^ and triply degenerated bending mode of phosphate (ν_4_PO_4_) at 561 cm^−1^ and 602 cm^−1^ [33]. FTIR spectrum shows bending (ν_2_CO_3_) and stretching (ν_3_CO_3_) vibration mode of carbonate that corresponds with B-type carbonated apatite (partial substitution of PO_4_^3−^ groups mainly by CO_3_^2−^ ions) [33]. The formation of nanocrystalline apatite was confirmed by XRD. Figure 3c (inset) exhibits broad reflections ascribed to hydroxyapatite (HA, ASTM card file No 09-432). The broadness of the peaks indicates low crystallinity and nanosized dimensions of the diffracting crystal domains. The ratio of mineral content of RCP.MgAp scaffold was quantitatively assessed by TGA of the top, middle and base of the scaffold to evaluate the mineral distribution (Figure 3c). TGA curves mainly exhibit three decomposition steps: the first loss from 25 °C to 170 °C due to the decomposition of adsorbed and bound water (accounting for 6–7 wt.%); the second loss from 170 °C to 420 °C due to degradation of recombinant collagen; and the third loss from 420 °C to 650 °C attributed to the combustion of organic residues. The resultant residue after 650 °C corresponds directly to the inorganic phase amount, which was 26 wt.% at the top and around 29 wt.% at the middle and base of the scaffold. This finding confirms a homogeneous distribution of Ap along the scaffold. Chemical composition of hybrid scaffolds was quantitatively evaluated by inductively coupled plasma optical emission spectroscopy (ICP-OES) indicating the following composition: Ca (9.4 ± 0.8 wt.%), PO_4_ (12.0 ± 1.4 wt.%) and Mg (0.8 ± 0.1 wt.%).

### 2.2. In Vitro Studies

#### 2.2.1. Optimization of hMSC Seeding on Scaffolds

**Figure 4:**
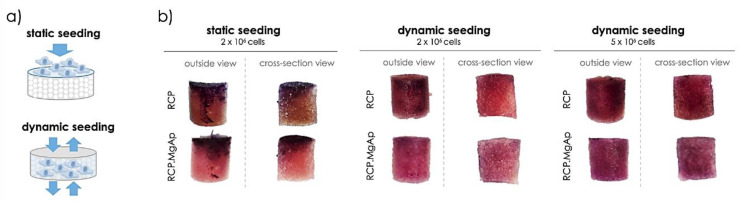
Cell seeding optimization. (**a**) Schematic design of the cell seeding approaches: static seeding in a well-plate and dynamic seeding in a perfusion bioreactor. (**b**) Representative images of MTT staining obtained after 24 h of hMSC culture on mineralized (RCP.MgAp) and non-mineralized (RCP) scaffolds. Full scaffolds were cross-sectioned in half in order to assess the cell distribution also in the center of the scaffold.

Cell seeding was initially optimized in order to achieve a homogeneous distribution of cells over the scaffold volume. First, 2 × 10^6^ cells were seeded on top of the scaffolds and maintained in static culture for 24 h (Figure 4a). This strategy led to the formation of cell clusters at the top and the edges of both mineralized (RCP.MgAp) and non-mineralized (RCP) scaffolds (Figure 4b). To overcome this undesired cell distribution, perfusion bioreactor was used for dynamic cell seeding using the same cell density (Section 4.4.3). The homogeneity of cell distribution within the scaffold improved significantly after 24 h of dynamic cell culture. Nonetheless, the MTT staining showed low cell density (pale violet) likely due to the large volume of the scaffolds (Figure 4b), giving rise to low MTT absorbance value (Supplementary Figure S2). The increase of cell suspension density to 5 × 10^6^ cells per scaffold allowed to obtain a homogeneous cell distribution with higher cell density through the whole volume, as shown MTT staining images of the lateral surface and the cross-section of the scaffolds (Figure 4b) and MTT absorbance graph (Supplementary Figure S2). These conditions were then used to further evaluate the capability of RCP.MgAp scaffold in inducing osteogenic differentiation of hMSC using a perfusion bioreactor.

#### 2.2.2. Dynamic Osteogenic Differentiation in a Perfusion Bioreactor

**Figure 5:**
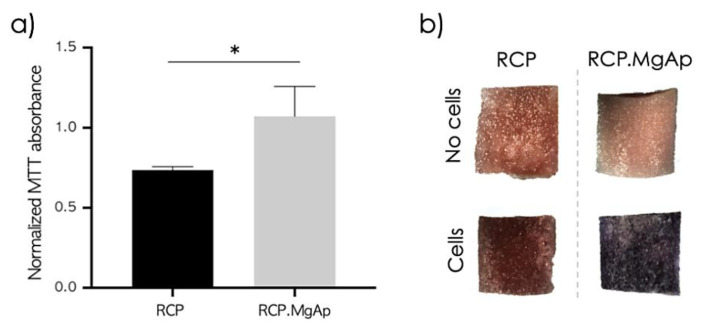
Cell viability of hMSC after 15 days of dynamic osteogenic culture. (**a**) MTT absorbance from full scaffolds was measured and normalized with dimethyl sulfoxide blank values. Statistical analysis using student *t*-test, * *p* < 0.05, *n* (RCP) = 3, *n* (RCP.MgAp) = 5. (**b**) Representative cross-sections images from MTT staining of non-seeded scaffolds (on top) and hMSC seeded scaffolds (on bottom) after 15 days of dynamic culture with osteogenic differentiation factors.

**Figure 6:**
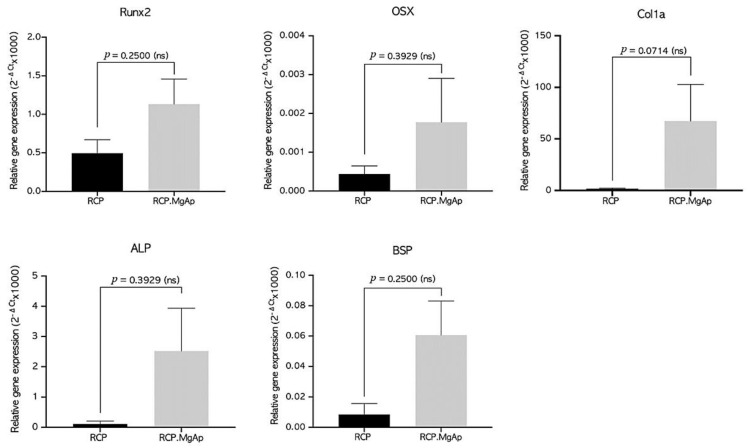
Relative gene expression (2^−ΔCt^) after 15 days. GAPDH housekeeping gene was used to normalize data. RUNX2 = runt-related transcription factor 2, ALP = alkaline phosphatase, Col1a = collagen type I alpha 1, OSX = osterix, BSP = bone sialoprotein. Statistical analysis using student *t*-test, *n* (RCP) = 3, *n* (RCP.MgAp) = 5.

The impact of the mineral phase on osteogenic differentiation of hMSC was studied after 15 days of dynamic cell culture in the perfusion bioreactor. At the end of the experiment, scaffolds were stained with MTT to assess cell viability and distribution over the scaffolds (Figure 5) and cells were collected for gene expression analysis (Figure 6). Interestingly, the presence of mineral provides a significant increase on cell viability for hMSC on RCP.MgAp scaffolds in comparison to the non-mineralized RCP scaffolds (Figure 5a). However, it was observed a homogeneously distributed staining for both materials (Figure 5b). As expected, the not-seeded scaffolds (control) showed no staining. Therefore, the increase of MTT absorbance is assumed to be associated with an increase of hMSC proliferation on mineralized scaffolds, rather than a hitch from the RCP scaffolds.

Regarding the gene expression analysis, hMSC cultivated on RCP.MgAp scaffolds showed the trend of superior osteogenic gene expression than hMSC cultivated on scaffolds without mineral (RCP) (Figure 6). Remarkably, the expression of Col1a1 on RCP.MgAP scaffolds showed a considerable increase in comparison to the control. Despite this clear trend, statistical analysis indicated no significance (*p* > 0.05). These results are indicative of an augmented differentiation into osteogenic lineage of hMSC cultivated in the mineralized scaffolds.

## 3. Discussion

The synergy of 3D scaffolds and perfusion bioreactors represents a promising strategy to more accurately simulate growth environment of stem cells in the human body [34]. In bone tissue engineering, 3D scaffolds must be designed to provide a more conductive structure for hMSC attachment, growth and osteogenic differentiation [34]. Herein, bioinspired scaffolds mimicking the osteogenic niche of cancellous bone have been designed through biomimetic mineralization of recombinant collagen and freeze-drying. This technique is one of the most common methodologies used to fabricate tridimensional porous scaffolds with tailored pore size, porosity and pore distribution (aligned or isotropic structures) [10,35]. These are critical parameters that directly affect the fluid shear stress sensed by the cells when cell medium is perfused through the scaffold in the bioreactor [36]. This mechanical stimulus has been pointed out as a potent promoter of hMSC osteodifferentiation [27,37]. A recent review suggests that the cultivation of hMSC under oscillatory or pulsative flow in 3D scaffolds (100–800 μm) and in the presence of osteoinductive supplements provides optimal effect on hMSC osteogenesis [23]. In this line, we obtained 3D scaffolds with pore size distribution (50–700 μm) inside the range previously commented. Moreover, the most frequent value of pore diameter (100–300 μm) has been designated as the optimum cell migration and new bone formation [38,39,40]. The porosity, closed to that found in cancellous bone, also provides a suitable structure for cell colonization and fluid exchanges [38,39,40]. Conversely, porosity has a detrimental effect on the scaffold mechanical properties, as reviewed by Deville et al. [35]. Future work, also involving in vivo studies, will require a thorough study to achieve a compromise between the requirements of porosity for cell ingrowth and fluid exchange and the need of suitable mechanical properties.

Biomimetic mineralization of RCP in the presence of magnesium promoted the nucleation of Ap nanoparticles into the organic matrix providing a homogeneous integration of the mineral phase along the entire scaffolds, as indicated by EDS map and TGA analysis. SEM image of the wall of the pores confirmed the formation of acicular nanoparticles in the organic matrix. These results fit with previous atomic force microscopy observations revealing a homogeneous distribution of Ap nanoparticles into the RCP matrix at nanoscale level when mineralization was carried out in the presence of Mg [20]. Conversely, RCP mineralization in the absence of Mg prompted to Ap nanoparticle aggregation, heterogeneous mineral distribution at nanoscopic scale and therefore, higher surface roughness value [20]. Regarding the chemical composition, these nanoparticles contain magnesium (close to the values found in bone, 0.5–1%) [21] and carbonate ions substituting phosphate group in apatite crystal structure, named as B-type carbonate substitution. This type of carbonate substitution is the preferred in bone apatite [41]. Whereas carbonate ions are by far the most abundant exogenous ion of bone apatite [41], magnesium is one of the more important minor cations in bone tissue [42]. Both ions, magnesium and carbonate, are typically found in younger bone triggering to poor crystalline apatite [14,43]. In fact, XRD pattern of RCP.MgAp scaffolds confirmed the precipitation of poor crystalline apatite as biological ones [12].

In addition to biomaterial features, cell distribution, mass transport and biomechanical stimulation are also highly relevant factors on bone regeneration [44]. In this scenario, perfusion bioreactors have been demonstrated to provide a homogeneous distribution of cells inside 3D scaffolds, an adequate transport of nutrients and oxygen and mechanical stimuli by way of fluid shear stress [26,44,45]. In this work, hMSC have been cultured on mineralized recombinant collagen scaffolds in the presence of Mg in a perfusion bioreactor in order to simultaneously provide them with the adequate physic-chemical and mechanical stimuli for osteogenic differentiation. The first step was to optimize hMSC seeding on 3D hybrid scaffolds. Whereas some authors carried out cell seeding in static conditions and then moved cell/scaffold construction inside the bioreactor for dynamic culture [46,47,48,49], others studies implemented dynamic conditions for cell seeding and culture [30,50,51]. Static cell seeding is by far the most commonly used seeding method but it provides low seeding efficiencies and non-uniform cell distribution [52]. Herein, the static cell seeding promoted the formation of capsule cell layer at the surface of the scaffold (closer to 2D system) due to the physical limitation of fluid penetration extensively reported in the literature, especially for bone substitutes constructs where a large scaffold volume is often required [53,54,55]. The dynamic seeding provided a more uniform cell distribution in both scaffolds, mineralized (RCP.MgAp) and non-mineralized ones (RCP, control), revealing their feasibility to support cell attachment. After 15 days of dynamic cell culture, mineralized scaffolds showed a significant increase on cell growth and scaffold repopulation comparing with non-mineralized scaffolds. This effect may be associated to the scaffold surface chemistry and topography [6]. Respect to the former, apatite nanoparticles are widely considered as ideal biomaterial for bone regeneration due to its excellent biocompatibility, biodegradability and osteoconductive and osteoinductive potential [5]. Moreover, the presence of magnesium may favor cell proliferation on mineralized scaffolds since magnesium is involved in bone homeostasis by enhancing attachment and differentiation of osteoblastic cells and accelerating the mineralization process [56]. Another factor to consider is the unique surface properties of nanocomposite materials that are capable of mediating specific protein adsorption and promoting cell adhesion, proliferation and differentiation [6]. In fact, a clear trend for upregulation of genes related to osteogenic commitment of primary hMSC cultured in mineralized scaffolds (RCP.MgAp) when compared with non-modified scaffolds (RCP) was observed. Despite this trend, no statistically significant differences in the expression of the tested genes were observed due to variations between donors, a fact widely reported and accepted in the literature for primary cells derived from different patients [57]. Moreover, the presence of osteogenic factors (L-ascorbic acid, dexamethasone and β-glycerophosphate) also induced dramatic differences in levels of bone-specific gene induction in hMSC exposed to osteo-inductive media, as previously reported by Donald G. Phinney et al. [58]. Yet, a prominent upregulation of Col1a1, an early precursor protein responsible for the formation of very strong mature type I collagen fibers, was observed for RCP.MgAp scaffolds.

In a nutshell, the biomimetic mineralization of RCP in the presence of magnesium might provide chemical signaling favoring hMSC viability and osteogenesis while perfusion bioreactor enhances cell distribution inside the scaffolds, closely recreating 3D bone ECM environment. Further studies are required in order to verify the osteogenic potential of mineralized recombinant collagen scaffolds. In addition, this work paves the way on the development of in vitro models that better recapitulate the in vivo cell niche, which are essential for the progress of fundamental biology and clinical translation (review in [59]).

## 4. Materials and Methods

### 4.1. Materials

Recombinant collagen peptide (RCP), commercially available as Cellnest™ (Fujifilm, Tilburg, The Netherlands), is produced by a fermentation process using genetically modified yeast as described previously [60]. RCP sequence is based on human collagen type I and comprises 571 amino acids. Unless noted, all reagents were purchased from Sigma-Aldrich (Taufkirchen, Germany).

### 4.2. Fabrication of Hybrid Scaffolds

Mineralization of RCP was carried out by a neutralization reaction in order to obtain a hybrid scaffold with a 30 wt.% of mineral content (i.e., magnesium-doped hydroxyapatite). Briefly, 0.75g of RCP was dissolved in 5 mL of phosphoric acid aqueous solution (270 mM) through magnetically stirring at 40 °C. Once that RCP was completely dissolved, it was added dropwise into 5 mL of a calcium hydroxide aqueous suspension (450 mM) containing magnesium ions in a molar ratio Mg/Ca of 0.15. After 2 h of mineral maturation at room temperature, the mineralized solution was poured into a 48-well plate, frozen at −20 °C overnight and freeze-dried (0.1 mbar; 5Pascal; Cinquepascal srl, Trezzano sul Naviglio, Italy). RCP aqueous solution (7.5 wt.%) was also freeze-dried in 48-well plate to fabricate control scaffolds for in vitro experiments. We obtained cylindrical scaffolds with a diameter of 10 mm and a height of 10 mm. The scaffolds were crosslinked by dehydrothermal treatment (DHT) at 160 °C under vacuum for 48 h. Finally, the scaffolds were sterilized by autoclaving.

### 4.3. Characterization of the Material

#### 4.3.1. Structural Characterization

The porosity of the scaffold and its three-dimensional structure was determined by X-ray computerized axial microtomograph (Zeiss Xradia 510 Versa, Oberkochen, Germany) of the Centre for Scientific Instrumentation (University of Granada, CIC-UGR). For each scaffold (d = 4 mm, h = 4 mm), 2000 radiographic images (image resolution: 5 μm, X-ray energy: 80 keV, exposure time = 3s) were obtained. ImageJ software (version 1.48v; NIH, Bethesda, MD, USA) was used to analyze pore size distribution and porosity in 200 images. The 3D image reconstruction of the scaffold was performed with Dragonfly Pro (Object Research System, Montreal, QC, Canada).

Scaffold structure and composition was analyzed with a Field Emission Scanning Electron Microscope (FESEM, Zeiss SUPRA40V from CIC-UGR) equipped with an Oxford energy-dispersive X-ray spectrophotometer (EDS) and operating at 3–15 KeV. Scaffolds were cut with a razor blade, mounted on aluminum stubs using a carbon tape and sputtered with a thin carbon film. Mineral distribution and composition were analyzed with Aztec 3.0 SP1 EDS software.

#### 4.3.2. Characterization of Chemical Composition

Fourier-transform infrared (FTIR) spectrum of RCP.MgAp scaffolds was recorded on a Nicolect 380 FTIR spectrometer (Thermo Fisher Scientific, Waltham, MA, United States of Ameria). Scaffold was cut with a razor blade in a small piece of 5 mg that was mixed with 250 mg of potassium bromide (KBr). Then, the mixture was pressed at 10 tons into 7 mm diameter disk. A pure KBr disk was used as blank. The spectrum was registered from 4000 cm^−1^ to 400 cm^−1^ with a resolution of 2 cm^−1^ by accumulating 100 scans.

Thermogravimetry analysis was performed using a simultaneous thermal analyzer (STA 449 Jupiter Netzsch Geratebau, Selb, Germany) with a heating rate of 10 °C/min up to 1000 °C in nitrogen flow.

X-Ray diffraction (XRD) pattern of RCP.MgAp scaffolds was recorded in a D8 Advance diffractometer (Bruker, Germany) using a Cu Kα radiation (λ = 1.5418 Å) generated at 40 kV and 40 mA. The pattern was recorded from 10° to 60° (2θ) with a step size of 0.002° and a scan rate of 5 s step^−1^.

The chemical composition (Ca, P and Mg) was analyzed by inductively coupled plasma optical emission spectrometry (ICP-OES; Agilent Technologies 5100 ICP-OES, Santa Clara, CA, USA). A 20 mg portion of scaffold was dissolved in 2 mL of ultrapure nitric acid and then diluted up to 100 mL with Milli-Q water. Three replicates were prepared and the Ca, P and Mg content were measured three times each replicate at analytical emission wavelengths of 422 nm (Ca), 279 nm (Mg) and 214 nm (P).

### 4.4. In Vitro Studies in Dynamic Conditions

#### 4.4.1. Cell Isolation and Expansion

hMSC were isolated from human bone marrow of femoral heads after informed consent of the patient and ethical approval (186/18), as previously described [61]. Then, hMSC were expanded in DMEM GlutaMAX medium (Gibco, Dreieich, Germany) supplemented with 10% FCS (Bio&Sell, Feucht, Germany), 1% Pen/Strep (Sigma-Aldrich), 1% HEPES (Sigma-Aldrich) and 5 ng/mL of recombinant human fibroblast growth factor (FGF, PeproTech, Hamburg, Germany) at 37 °C in a humidified atmosphere of 5% CO_2_. Culture medium was replaced 3 times a week. Cells were detached from culture flasks by trypsinization, centrifuged and resuspended. Cell number and viability were assessed with the trypan-blue dye exclusion test. hMSC in passage 4–6 were used for the experiments.

hMSC characterization of these cell population, such as surface markers analysis and in vitro osteogenic and adipogenic potential, have been proven in ref. [62].

#### 4.4.2. Perfusion Bioreactor

**Figure 7:**
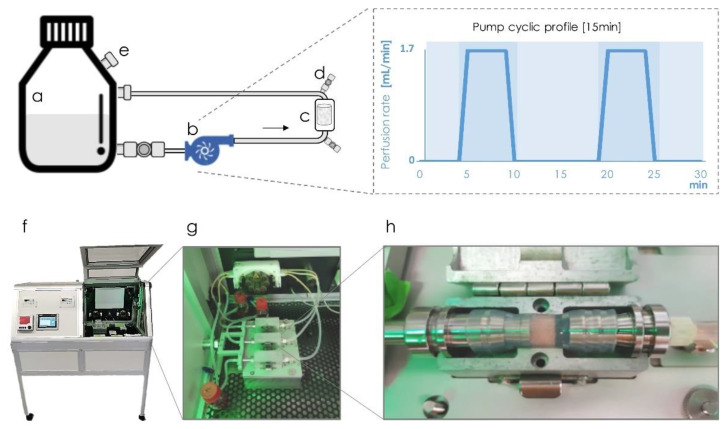
Schematic representation of the bioreactor design. The cell culture media is kept in a controlled environment at 37 °C in a flask reservoir (**a**) and driven through silicon tubes with a controllable pump (**b**) towards the scaffold chamber (**c**,*h*) and back to the reservoir. The system includes additional connections (**d**) strategically in the inlet and outlet of the chamber for cell seeding. A valve equipped with a sterile filter (**e**) on the reservoir guarantees the gas exchange with the external environment. The pump is programmed to actively perfuse cell culture medium for 5 min at a rate of 1.7 mL/min and stop for 10 min in cycles of 15 min, as illustrated on the right graph. The incubation chamber (**f**) includes temperature and CO_2_ control where multi bioreactor systems can be mounted in parallel (**g**).

This perfusion bioreactor consists of a computer-controlled pump connected by silicon tubes to a scaffold chamber and a flask reservoir in a closed cyclic system (Figure 7). The scaffold chamber is sealed with silicone tube with an inner diameter of 10 mm, the same diameter as the RCP.MgAp scaffolds (Figure 7h). The scaffold chamber was mounted on a bioreactor cartridge that ensures the homogeneous conditions, avoiding the extravasation of overflow out of the system, while allowing gas exchange with the bioreactor environment. The bioreactor was maintained in aseptic conditions in a controlled chamber (Figure 7f) at 37 °C and with 5% CO_2_. This bioreactor has been satisfactorily used for dynamic culture of hMSC on synthetic polymeric scaffolds (non-mineralized) to evaluate its osteogenic differentiation with or without external mechanical stimulation as deformation strain [50,63].

#### 4.4.3. Optimization of hMSC Seeding in 3D Scaffolds

Non-mineralized RCP scaffolds were used as a control for in vitro test. All scaffolds (10 mm in diameter and 10 mm of height) were pre-incubated with complete culture medium at 37°C overnight before cell seeding.

Two different strategies (static and dynamic) of cell seeding were evaluated. For static seeding, scaffolds were placed in a 24-well plate and each seeded by carefully dropping 100 μL of cell suspension containing 2.0 × 10^6^ cells onto the upper surface, allowing cell attachment for 90 min at 37 °C with 5% CO_2_. Then, 1.5 mL of expansion medium supplemented with 10 mM β-glycerophosphate (Sigma-Aldrich), 50 µg/mL L-ascorbic acid (Sigma-Aldrich) and 100 nM dexamethasone (Sigma-Aldrich) were added to each well and hMSC were cultured for 1 day.

For dynamic seeding, the scaffolds were placed in the bioreactor chamber and then, 750 μL of cell suspension containing 2.0 × 10^6^ or 5.0 × 10^6^ cells were manually inserted in the bioreactor chamber through connection named as d (Figure 7). After five backward-forward cycles, cells were allowed to adhere for 90 min at 37 °C with 5% CO_2_. Then, the complete supplemented culture medium (35 mL) was added to the reservoir flask and driven through silicon tubes (towards the scaffold chamber and back to the reservoir) at a rate of 1.7 mL/min for 5 min every 15 min for 1 day.

Cell viability and distribution in the scaffolds were assessed with MTT [3-(4,5-dimethylthiazol-2-yl)-2,5diphenyltetrazolium bromide] assay. Briefly, cell-laden scaffolds were incubated for 2 h in a solution of 10% MTT (5 mg/mL) in cell culture medium. The metabolically active cells react with the tetrazolium salt in the MTT reagent producing a formazan dye. Pictures of scaffold cross-section were taken under a stereomicroscope (Zeiss, Discovery.V20).

#### 4.4.4. Osteogenic Differentiation of hMSC under Flow Perfusion

5.0 × 10^6^ cells were seeded and cultured under dynamic conditions (as previously described in Section 4.4.2) for 15 days to evaluate the osteogenic differentiation. Media change was carried out 3 times per week. A total of 3 donors were used as biological replicas for RCP control samples, while 5 hMSC donors were tested for RCP.MgAp scaffolds.

Cell viability was evaluated by MTT assay (see above). After taking some pictures, scaffolds were transferred to a tube containing dimethyl sulfoxide (DMSO) that dissolves formazan crystal and absorbance was read at 570 nm using a Tecan’s NanoQuant plate reader.

The osteoblastic differentiation was evaluated by analyzing the expression of osteogenic genes by real-time polymerase chain reaction (RT-qPCR). Scaffolds were homogenized in 1 mL of TRI Reagent (Sigma-Aldrich) for 5 min at 50 Hz using a QIAGEN TissueLyser LT (Hilden, Germany). Supernatant was collected and RNA extracted according to the manufacturer’s protocol (NucleoSpin RNA, Macherey-Nagel, Dueren, Germany). cDNA was synthesized using Promega reverse transcriptase reagents. Real-time Polymerase chain reaction (PCR) was performed on the CFX96 Real-Time System (Bio-Rad, Postfach, Germany). Selected osteogenic marker genes were detected using Runx2 (NM 001015051.3), ALP (NM 001631.3), Col1a1 (NM 000088.3), OSX (NM 001173467.2), and BSP (NM 004967.3) primers, all purchased from Applied Biosystems (Dreieich, Germany). GAPDH (NM 002046.3) was used as a housekeeping gene. Non-mineralized scaffolds (RCP) were used as control. Relative quantification of mRNA targets was performed according to the comparative 2^−ΔCt^ × 1000 method [64].

### 4.5. Statistical Analysis

Results are expressed as the mean ± standard error of the mean (SEM). Data analysis was made by *t*-test, followed by non-parametric analysis using Mann–Whitney test. Statistical analyses were performed with the GraphPad Prism software (version 6.0) and statistical significance set at *p* < 0.05

## 5. Conclusions

In this study, the combination of mineralized recombinant collagen scaffolds in the presence of magnesium with dynamic culture of hMSC was investigated for the first time. 3D porous structure with suitable pore size and porosity of cell ingrowth was obtained via freeze-drying. Biomimetic mineralization in the presence of Mg provided a homogeneous mineral incorporation into the organic matrix. This mineral phase consisted of poor crystalline apatite nanoparticles containing carbonate and magnesium, two relevant ions found in bone apatite. Dynamic seeding of hMSC provided a more uniform cell distribution along the inner areas of the scaffolds compared to static cell seeding approach, thus resulting in a genuine 3D culture system. After 15 days of dynamic culture, mineralized scaffolds enhanced hMSC viability and showed a clear trend of enhancement of osteogenic differentiation compared to non-mineralized scaffolds, revealing the pivotal role of biomimetic mineral phase on cell behavior. Further studies are required in order to verify this hypothesis and optimize the system towards specialized tissue-engineered constructs for bone replacement.

## Data Availability

MDPI Research Data Policies.

## Supplementary Materials

The following are available online at https://www.mdpi.com/1422-0067/22/3/1447/s1, Figure S1: Structural characterization of non-mineralized collagen scaffolds (Control, RCP) by micro-computed tomography. Figure S2: MTT absorbance of cell seeding optimization experiment.

## Abbreviations

3D	Three-dimensional
ECM	Extracellular matrix
hMSC	Human mesenchymal stem cells
Ap	Apatite
RCP	Recombinant collagen peptide
RCP.MgAp	Mineralized recombinant collagen scaffold in presence of magnesium
μCT	Micro-computed tomography
SEM	Scanning electron microscopy
EDS	Energy dispersive X-ray spectroscopy
FTIR	Fourier-transform infrared spectroscopy (FTIR)
TGA	Thermogravimetric analysis
XRD	X-ray diffraction
MTT	3-(4,5-dimethylthiazol-2-yl)-2,5diphenyltetrazolium bromide
DMSO	Dimethyl sulfoxide
RUNX-2	Runt-related transcription factor
ALP	Alkaline phosphatase
Col1a	Collagen type I alpha
OSX	Osterix
BSP	Bone sialoprotein
