# Supplementary material for: Biomimetic Mineralization Promotes Viability and Differentiation of Human Mesenchymal Stem Cells in a Perfusion Bioreactor
Source: Int J Mol Sci. 2021 Feb 1;22(3):1447. doi: 10.3390/ijms22031447 (PMC7867135; doi:10.3390/ijms22031447)
Supplement: Supplementary file 1 [file ijms-22-01447-s001.pdf]

Supplementary information

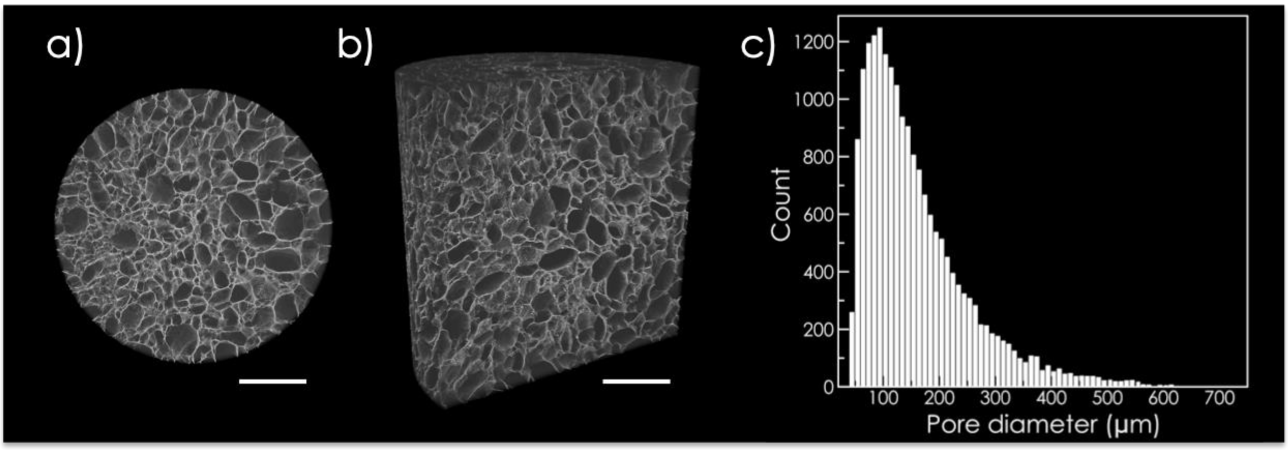

Figure 1. Structural characterization of non-mineralized collagen scaffolds (Control, RCP) by micro-computed tomography (diameter = 4mm; height = 4 mm). (a) Transversal and (b) longitudinal section of scaffold. Scale bar= 1 mm. (c) Graph bar display pore size distribution.

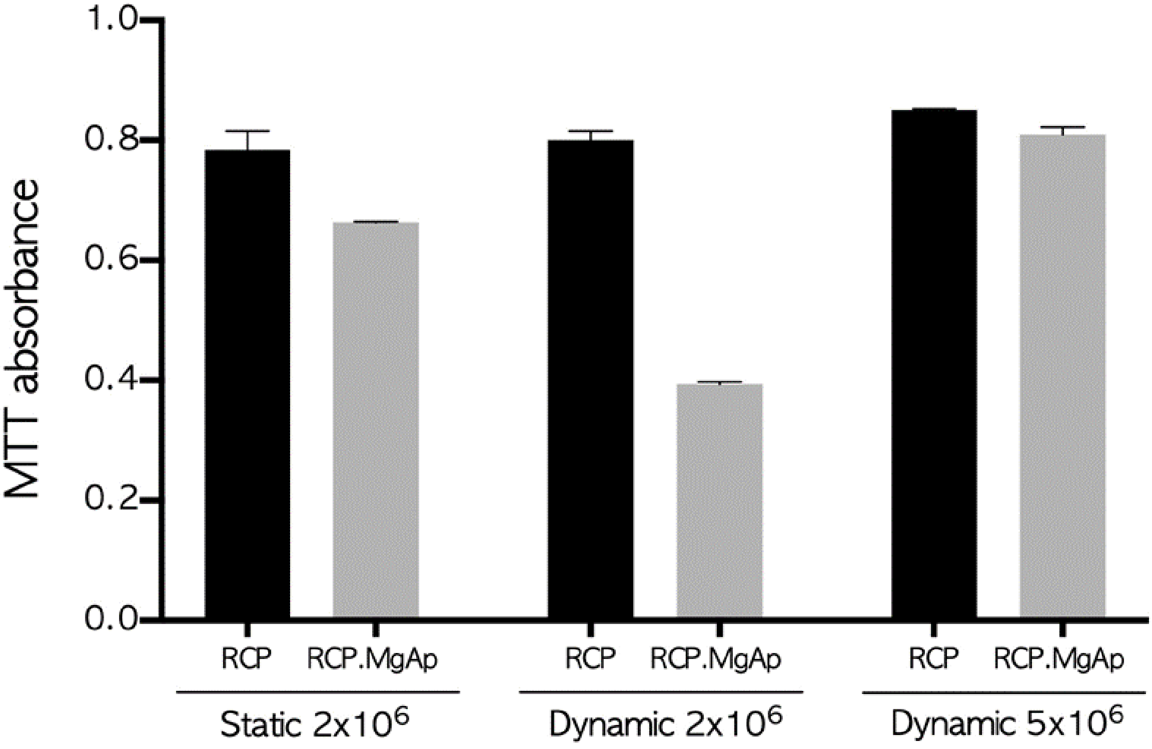

Figure S2. MTT absorbance of cell seeding optimization experiment. n=2 technical replicates. Mean ± SEM.
